# Changes in attitudes toward systemic lupus erythematosus and associated factors: a retrospective cross-sectional study from China

**DOI:** 10.3389/fimmu.2026.1759777

**Published:** 2026-01-26

**Authors:** Minjing Zhao, Si-Wei Xie, Xinxiang Huang, Baozhao Xie, Xianyi Zeng, Claire Chenwen Zhong, Zhiming Lin

**Affiliations:** 1 Department of Rheumatology, Third Affiliated Hospital of Sun Yat-sen University, Guangzhou, China; 2 Department of Rehabilitation, Nyingchi People’s Hospital, Nyingchi, China; 3 Johns Hopkins School of Medicine, Baltimore, MA, United States; 4 Department of Rheumatology and Immunology, The People’s Hospital of Guangxi Zhuang Autonomous Region, Nanning, China; 5 Department of Rheumatology and Immunology, The Seventh Affiliated Hospital of Guangxi Medical University, Guangxi, China; 6 China Unicom Digital Intelligence Medical Technology Co., Ltd., Guangzhou, China; 7 JC School of Public Health & Primary Care, Faculty of Medicine, The Chinese University of Hong Kong, Hong Kong, Hong Kong SAR, China

**Keywords:** disease management, patient attitude, patient knowledge, questionnaire survey, systemic lupus erythematosus

## Abstract

**Introduction:**

Patient attitudes toward systemic lupus erythematosus (SLE) play a critical role in disease management, yet their evolution through the treatment course remains understudied. This study aimed to map changes in patients’ SLE-related attitudes from diagnosis to post-treatment and to identify sociodemographic, clinical, and management-related factors associated with these attitudinal changes.

**Methods:**

We conducted a multicenter cross-sectional retrospective study involving 1,509 patients with confirmed SLE from 105 hospitals across China. Participants completed a structured questionnaire assessing attitudes toward SLE at diagnosis and post-treatment. Attitudes were categorized as positive or negative, and changes classified as better, worsen, or unchanged. Multivariable logistic regression analyses were applied to identify factors associated with initial attitudes, post-treatment attitudes, and attitudinal changes.

**Results:**

At diagnosis, 959 (63.6%) patients held a negative attitude, while 550 (36.5%) reported a positive attitude. After treatment, 1,367 patients (90.6%) reported a positive attitude. At diagnosis, female sex and higher education level were associated with lower odds of a positive attitude, while familiarity with national SLE guidelines and doctor-oriented medication management were positively associated. After treatment, improved attitudes were significantly associated with older age, guideline familiarity, and collaborative management expectations. Conversely, younger onset age, side effects, and patient-led medication management were linked to persistent negative attitudes. Among patients with better attitudinal change, older age, higher education, guideline familiarity, and doctor-oriented and patient-involved management expectations were key predictors. Attitudes worsening was significantly associated with the incidence of extra side effects.

**Discussion:**

Patient attitudes toward SLE are dynamic and shaped by clinical experiences, knowledge, and treatment interactions. Recommendations include enhancing physician-patient interactions, strengthening patient education, and proactive intervention and management of treatment, particularly during early stages, to foster more positive attitudes and support better long-term outcomes.

## Introduction

Systemic lupus erythematosus (SLE) is a complex autoimmune disorder characterized by diverse clinical manifestations, challenging disease management, and substantial impact on quality of life for patients (1–3). Effective management of SLE typically necessitates long-term adherence to medications and ongoing patient involvement, both of which can be influenced by patients’ knowledge and attitudes toward SLE and its treatment (4, 5). Previous studies underscored the importance of patient adherence and attitudes in determining treatment efficacy and clinical outcomes (6–8). Patient knowledge regarding disease-specific guidelines, such as the familiarity of 2020 Chinese guidelines for the diagnosis and treatment of systemic lupus erythematosus among Chinese SLE patients (9), would also influence the treatment decisions, disease management, and health outcomes (10, 11). Given the chronic nature of SLE, further understanding the relationship between patient knowledge, attitudes, and treatment decisions remains a crucial challenging aspect of disease management.

Despite increasing recognition of the role attitudes and knowledge play in chronic disease management, notable gaps remain within current research. Most existing studies have focused primarily on cross-sectional analyses, neglecting longitudinal shifts in patient attitudes and knowledge throughout the treatment course (12, 13). Moreover, existing research rarely addressed the determinants of attitude shifts or how initial attitudes toward SLE might influence subsequent treatment adherence and management preferences. There is also a lack of consideration for variations in education, socioeconomic status, and healthcare practices among SLE patients, which could uniquely influence attitudes and knowledge (14, 15). Thus, identifying factors contributing to initial attitudes toward SLE and subsequent changes in these attitudes is crucial to developing tailored management interventions.

To fill these gaps, this retrospective cross-sectional study aimed to analyze the impact of patient attitudes toward to SLE on treatment decisions and disease management among Chinese patients diagnosed with SLE. This study sought to (1) identify factors associated with positive versus negative attitudes at the stage of diagnosis and post-treatment (after receiving treatment) separately (2), assess factors influencing attitudinal changes toward SLE after receiving treatment vs initial attitudes at diagnosis, and (3) evaluate the role of patient knowledge regarding SLE management guidelines on attitudes toward SLE. By assessing these associations, this study intends to inform targeted interventions to enhance patient adherence, optimize disease management strategies, and ultimately improve clinical outcomes for patients with SLE.

## Materials and methods

### Study population

In this retrospective cross-scetional study, we involved data collection from 1509 patients diagnosed with SLE across 105 hospitals in China. Information was gathered using a structured, questionnaire-based approach, implemented via an online registry platform to enhance accessibility and patient engagement. The survey was distributed through QR codes displayed at each participating site. Data were collected between June and December 2021. During this time, trained hospital staff or physicians assisted patients in accurately completing the questionnaires, helping to minimize errors and misreporting. Eligible participants were those with a confirmed diagnosis of SLE, regardless of disease stage.

### Measurement of attitude toward SLE

Attitude was assessed with a single survey item (Q43, **Supplementary Material**): “How has your attitude towards the disease changed from diagnosis to treatment?” Responses were: (1) From fear to fear; (2) From fear to acceptance; (3) From acceptance to fear; (4) From acceptance to acceptance.

Derivation of attitude variables:

- Attitude at diagnosis: “From acceptance to acceptance” and “From acceptance to fear” (options 3 & 4) = Positive; “From fear to acceptance” and “From fear to fear” (options 1 & 2) = Negative.
- Attitude post-treatment: “From acceptance to acceptance” and “From fear to acceptance” (options 2 & 4) = Positive; “From acceptance to fear” and “From fear to fear” (options 1 & 3) = Negative.
- Attitudinal change: Better = option 2; Worsen = option 3; No change = options 1 or 4.

### Outcomes

(1) Patients’ attitudes toward SLE: positive vs negative.

(2) The changes in patients’ attitudes toward SLE after receiving treatment vs initial attitudes at diagnosis. We defined this change as: better, worsen or no change.

### Variable selection

The variable we selected to assess association with attitude toward SLE included demographics (sex, age, education (primary middle school or below; high school; bachelor degree or above), monthly income (¥) (<3000; 3000-5000; 5000-10,000; >10,000), medical history and clinical data (onset age, chronic diseases before the diagnosis of SLE, development of chronic diseases during treatment (no change; increase one chronic disease; increase two chronic diseases; increase three chronic diseases or more), assessed via patient self-report from a prespecified list (e.g., hypertension, diabetes; see **Supplementary Material** for full list), development of side effects related the drugs during treatment (no change; increase one extra side effect; increase two extra side effects; increase three extra side effects), assessed via patient self-report from a prespecified list (e.g., femoral head necrosis, serious infection; see **Supplementary Material** for full list), medications (use of immunosuppressive drugs in the initial medication, use of immunosuppressive drugs in the existing medication, use of biological agents in the initial medication, use of biological agents in the existing medication, changes in medication (No change; change one time; change twice; change triple times)), attitude toward SLE, knowledge of the treatment guidelines (understanding of SLE guideline of 2020 Chinese guidelines for the diagnosis and treatment of systemic lupus erythematosus (not at all; a little bit; familiar or understand), and treatment management-related variables (initial medication management approach (doctor oriented; patient involved; self-initiated reduction of medication), existing medication management approach, patient expected medication management approach (patient oriented; doctor oriented; patient involved; self-initiated reduction of medication)) (4, 5, 9–11, 14–21).

### Time variables

Participants reported the date of their initial SLE diagnosis and the date they commenced the first SLE-specific treatment. The time from diagnosis to survey participation and the time since treatment initiation were calculated accordingly. These time intervals are reported as median and interquartile range (IQR) in the descriptive analyses.

### Statistical analysis

Data were collected via an electronic questionnaire system in which all survey items were mandatory; participants could not submit the questionnaire with any unanswered items. For descriptive comparisons, the data was divided by negative and positive attitude. We used the Shapiro-Wilk test to check whether continuous variables followed a normal distribution. When the data met normality assumptions, independent t-tests were used to compare groups. If the distribution was not normal, the Wilcoxon rank-sum test was chosen instead. For categorical variables, Fisher’s exact test was applied in cases with small sample sizes (usually fewer than 5 observations per categorical type), while chi-square tests were used when the sample size was larger.

We performed a series of multivariable logistic regression models to examine the relationship between selected factors and attitudes at different stages (22). In our analysis, separate models were developed for three key scenarios: (1) initial attitudes at diagnosis, (2) post-treatment attitudes (after receiving treatment), and (3) attitudes changes toward SLE after receiving treatment vs initial attitudes at diagnosis. Across all models, adjusted odds ratios (aORs) were reported along with their corresponding 95% confidence intervals, which serve as an index of the precision and uncertainty of our estimates (23). For attitudes assessed at the time of diagnosis and post-treatment, binary logistic regression models were constructed with positive vs negative attitude as the outcome. To explore factors influencing the direction of attitudinal change, we conducted two logistic regressions with “no change” as the reference category, modeling separately the factors of “worsened” and “better” attitudes. These analyses allowed us to examine the extent to which (baseline) characteristics, clinical status, and management expectations contributed to changes in attitudes toward SLE.

All data cleaning, preprocessing, and statistical analyses were performed using the R software (version 4.4.2, R Project for Statistical Computing). A two-tailed p-value threshold of less than 0.05 was applied to determine statistical significance.

## Results

### Time from diagnosis and treatment initiation

For the study cohort, the median time from diagnosis to survey participation was 4.00 years (IQR 2.00–8.00 years).

### Characteristics at the phase of diagnosis

**Table 1** showed the description of characteristics at the phase of diagnosis. A total of 959 patients (63.6%) had negative attitude toward SLE at the phase of diagnosis, while 550 patients (36.5%) showed positive attitude. Overall, the proportion of patients with a negative attitude was higher compared to those with a positive attitude. Females accounted for a higher percentage in the negative attitude group than in the positive group (94.8% vs 91.5%, *p* = 0.015). Patients with negative attitudes had a higher proportion of individuals with bachelor’s degrees or above compared with positive attitudes (46.0% vs 31.8%, *p* = 0.001), whereas the proportion with lower monthly incomes (<3000 RMB) was lower compared to patients expressing positive attitudes (44.8% vs 53.5%, *p* = 0.015). Additionally, a lower percentage of patients in the negative attitude group reported familiarity or understanding of the SLE guideline, compared with positive attitude group.

**Table 1:** Description of characteristics by attitude toward SLE at the phase of diagnosis.

Characteristics	Levels	Negative (N = 959)	Positive (N = 550)	P value
Sex, n (%)	Man	50 (5.2)	47 (8.5)	.015
	Female	909 (94.8)	503 (91.5)	
Age (years), mean (SD)		34.4 ± 9.8	35.4 ± 10.8	.078
Education, n (%)	Primary middle school or below	294 (30.7)	228 (41.5)	<.001
	High school	224 (23.4)	147 (26.7)	
	Bachelor degree or above	441 (46.0)	175 (31.8)	
Monthly income (¥), n (%)	<3000	430 (44.8)	294 (53.5)	.005
	3000-5000	344 (35.9)	181 (32.9)	
	5000-10,000	142 (14.8)	57 (10.4)	
	>10,000	43 (4.5)	18 (3.3)	
Onset age (years), mean (SD)	Mean ± SD	28.2 ± 9.8	28.6 ± 11.3	.395
Chronic diseases before the diagnosis of SLE, n (%)	Yes	134 (14.0)	69 (12.5)	.482
	No	825 (86.0)	481 (87.5)	
Use of immunosuppressive drugs in the initial medication, n (%)	Yes	702 (73.2)	395 (71.8)	.603
	No	257 (26.8)	155 (28.2)	
Use of biological agents in the initial medication, n (%)	Yes	52 (5.4)	25 (4.5)	.533
	No	907 (94.6)	525 (95.5)	
Understanding of SLE guideline, n (%)	Not at all	446 (46.5)	231 (42)	.019
	A little bit	378 (39.4)	212 (38.5)	
	Familiar or Understand	135 (14.1)	107 (19.5)	
Initial medication management approach, n (%)	Doctor oriented	883 (92.1)	487 (88.5)	.074
	Patient involved	65 (6.8)	54 (9.8)	
	Self-initiated reduction of medication	11 (1.1)	9 (1.6)	

**Figure 1:**
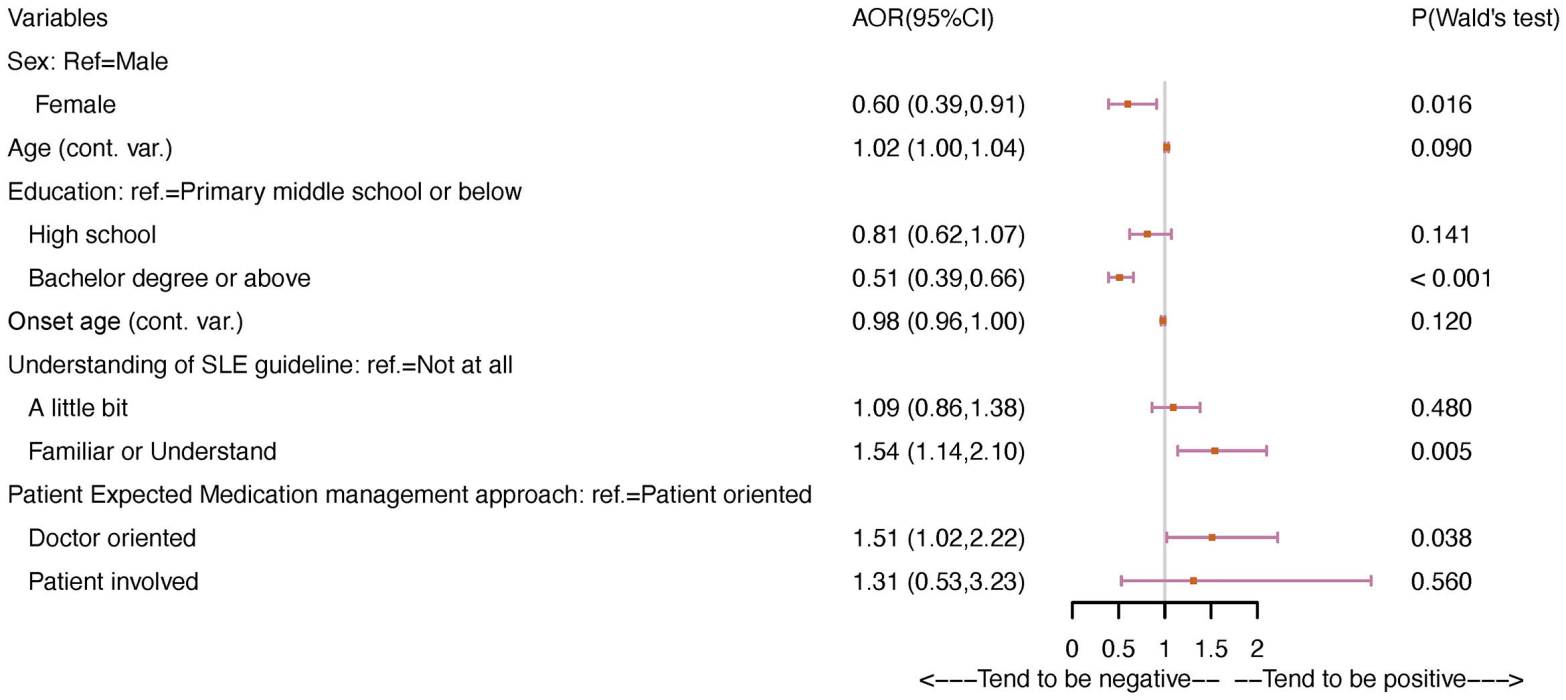
Associations between characteristics and attitudes toward SLE at the stage of diagnosis.

The following factors showed significant lower odds of having a positive attitude toward SLE: females vs males (aOR= 0.60, 95% CI: 0.39-0.91, *p* = 0.016), bachelor’s degree or higher education level vs primary middle school or below (aOR= 0.51, 95% CI: 0.39-0.66, *p<*0.001) (**Figure 1**). In contrast, the following factors had significant associations with higher odds of holding a positive attitude: familiar with or understood the SLE guidelines (aOR= 1.54, 95% CI: 1.14-2.10, *p* = 0.005), Doctor-oriented medication management vs patient-oriented management (aOR= 1.51, 95% CI: 1.02-2.22, *p* = 0.038).

### Characteristics at the phase of post-treatment

**Table 2** presented patient characteristics again according to attitudes toward SLE after receiving treatment. 142 patients (9.4%) reported a negative attitude, whereas a substantially larger number (1367 patients (90.6%)) held a positive attitude. Patients with positive attitudes had a higher proportion of individuals with bachelor’s degrees or above compared with negative attitudes (41.8% vs 31.0%, *p* < 0.001), whereas the proportion with lower monthly incomes (<3000 RMB) was lower compared to patients expressing positive attitudes (46.5% vs 62.7%, *p* < 0.001). These value distributions were different with that in the phase of diagnosis. In terms of side effects, a higher percentage of patients with negative attitudes reported more extra side effects both in initial and existing medication regimens, compared with proportion in positive patients (*p* = 0.004). A lower percentage of patients in the negative attitude group reported familiarity or understanding of the SLE guideline, compared with positive attitude group. Notably, patients who had negative attitudes at the time of diagnosis accounted for 47.9% of those negative after treatment, whereas among those who had positive attitudes post-treatment, a higher proportion of patients (65.2%) initially expressed negative attitudes at diagnosis (*p* < 0.001). After receiving treatment, there was a significant difference in the attitudes between the two groups with positive and negative attitudes at diagnosis (*p* < 0.001).

**Table 2:** Description of characteristics by attitude toward SLE post-treatment.

Characteristics	Levels	Negative (N = 142)	Positive (N = 1367)	P value
Sex, n (%)	Man	5 (3.5)	92 (6.7)	.192
	Female	137 (96.5)	1275 (93.3)	
Age (years), mean (SD)	Mean ± SD	33.6 ± 9.1	34.9 ± 10.3	.163
Education, n (%)	Primary middle school or below	69 (48.6)	453 (33.1)	.001
	High school	29 (20.4)	342 (25.0)	
	Bachelor degree or above	44 (31.0)	572 (41.8)	
Monthly income (¥), n (%)	<3000	89 (62.7)	635 (46.5)	.001
	3000-5000	40 (28.2)	485 (35.5)	
	5000-10,000	8 (5.6)	191 (14.0)	
	>10,000	5 (3.5)	56 (4.1)	
Onset age (years), mean (SD)	Mean ± SD	28.0 ± 9.2	28.4 ± 10.5	.669
Chronic diseases before the diagnosis of SLE, n (%)	Yes	18 (12.7)	185 (13.5)	.876
	No	124 (87.3)	1182 (86.5)	
Use of immunosuppressive drugs in the initial medication, n (%)	Yes	106 (74.6)	991 (72.5)	.653
	No	36 (25.4)	376 (27.5)	
Use of immunosuppressive drugs in the existing medication, n (%)	Yes	112 (78.9)	990 (72.4)	.121
	No	30 (21.1)	377 (27.6)	
Use of biological agents in the initial medication, n (%)	Yes	6 (4.2)	71 (5.2)	.765
	No	136 (95.8)	1296 (94.8)	
Use of biological agents in the existing medication, n (%)	Yes	11 (7.7)	97 (7.1)	.908
	No	131 (92.3)	1270 (92.9)	
Changes in medication, n (%)	No change	97 (68.3)	982 (71.8)	.695
	change one time	29 (20.4)	244 (17.8)	
	change twice	9 (6.3)	93 (6.8)	
	change triple times	7 (4.9)	48 (3.5)	
Development of chronic diseases during treatment, n (%)	No change	113 (79.6)	1167 (85.4)	.336
	increase one chronic disease	20 (14.1)	138 (10.1)	
	increase two chronic diseases	6 (4.2)	43 (3.1)	
	increase three chronic diseases or more	3 (2.1)	19 (1.4)	
Development of side effects during treatment, n (%)	No change	111 (78.2)	1172 (85.7)	.004
	increase one extra side effect	25 (17.6)	161 (11.8)	
	increase two extra side effects	3 (2.1)	30 (2.2)	
	increase three extra side effects	3 (2.1)	4 (0.3)	
Understanding of SLE guideline, n (%)	Not at all	81 (57.0)	596 (43.6)	.004
	A little bit	48 (33.8)	542 (39.6)	
	Familiar or Understand	13 (9.2)	229 (16.8)	
Existing medication management approach, n (%)	Doctor oriented	114 (80.3)	1169 (85.5)	.112
	Patient involved	23 (16.2)	177 (12.9)	
	Self-initiated reduction of medication	5 (3.5)	21 (1.5)	
Patient expected medication management approach, n (%)	Patient oriented	5 (3.5)	8 (0.6)	<.001
	Doctor oriented	98 (69.0)	961 (70.3)	
	Patient involved	35 (24.6)	384 (28.1)	
	Self-initiated reduction of medication	4 (2.8)	14 (1.0)	
Initial medication management approach, n (%)	Doctor oriented	122 (85.9)	1248 (91.3)	.070
	Patient involved	16 (11.3)	103 (7.5)	
	Self-initiated reduction of medication	4 (2.8)	16 (1.2)	
Attitude at the phase of diagnosis, n (%)	Negative	68 (47.9)	891 (65.2)	<.001
	Positive	74 (52.1)	476 (34.8)	

**Figure 2:**
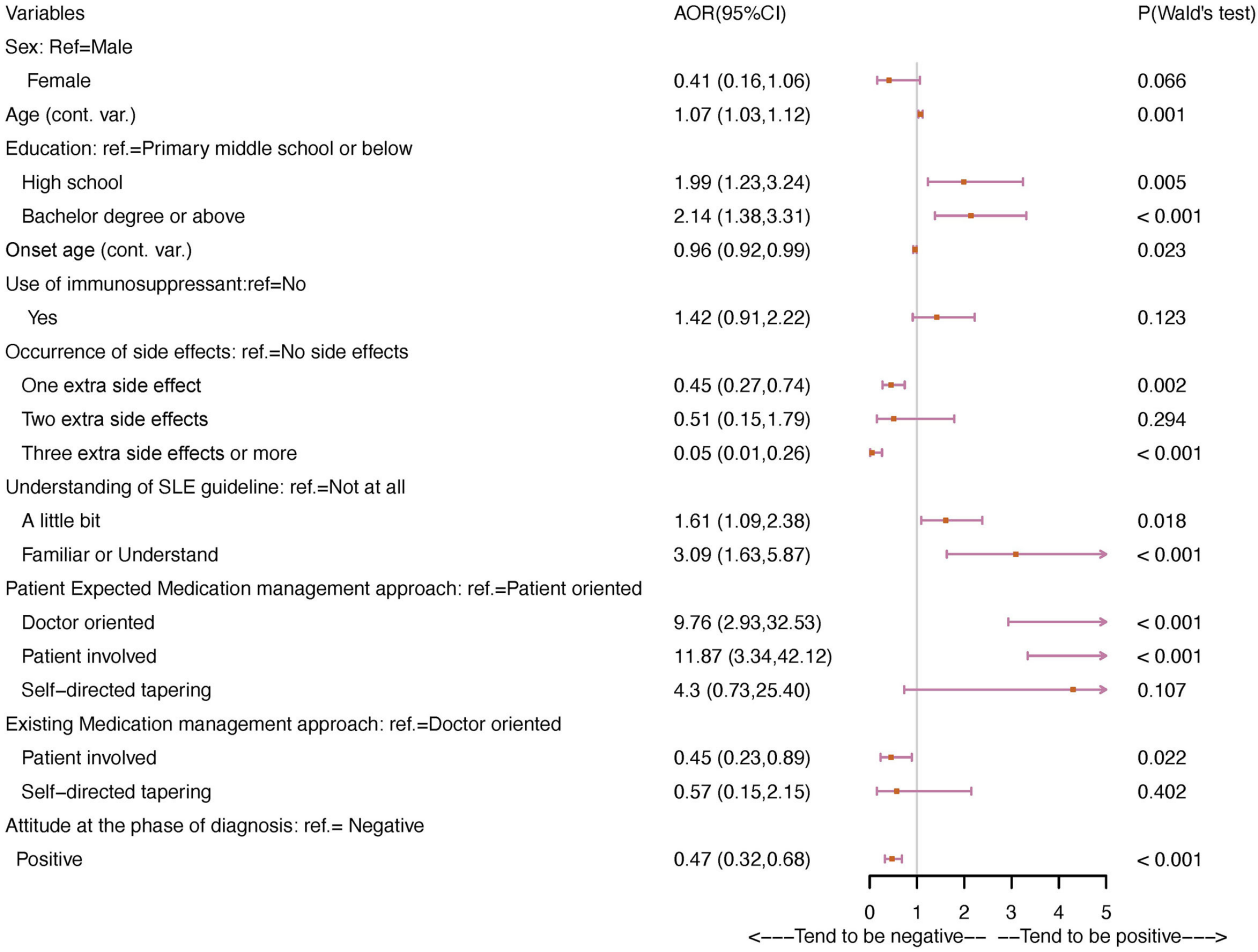
Associations between characteristics and attitudes toward SLE at the stage of post-treatment.

For attitude after receiving treatment, the following factors had significant associations with higher odds of holding a positive attitude: older age (aOR = 1.07, 95% CI: 1.03-1.12, *p* = 0.001), education level of high school (aOR = 1.99, 95% CI: 1.23-3.24, *p* = 0.005) or bachelor’s degree or higher (aOR = 2.14, 95% CI: 1.38-3.31, *p* < 0.001) compared to primary middle school or below, having a little bit (aOR = 1.61, 95% CI: 1.09-2.38, *p* = 0.018) or familiar understanding of SLE guidelines (aOR = 3.09, 95% CI: 1.63-5.87, *p* < 0.001), doctor-oriented (aOR = 9.76, 95% CI: 2.93-32.53, *p* < 0.001) or patient-involved (aOR = 11.87, 95% CI: 3.34-42.12, *p* < 0.001) medication management expectations compared to patient-oriented (**Figure 2**). In contrast, the following factors showed significant lower odds of having a positive attitude toward SLE after the phase of diagnosis: younger onset age (aOR = 0.96, 95% CI: 0.92-0.99, *p* = 0.023), experiencing one extra side effects (aOR = 0.45, 95% CI: 0.27-0.74, *p* = 0.002), three or more extra side effects (aOR = 0.05, 95% CI: 0.01-0.26, *p* < 0.001), patient-involved existing medication management vs doctor-oriented (aOR = 0.45, 95% CI: 0.23-0.89, *p* = 0.022), and positive attitude at the phase of diagnosis vs negative (aOR = 0.47, 95% CI: 0.32-0.68, *p* < 0.001).

### Change of attitudes toward SLE

**Table 3** described the changes in patients’ attitudes toward SLE after the phase of diagnosis. Among all patients, 74 individuals had the worsened attitudinal changes, 891 improved the better attitudinal changes, and 544 showed no change in their attitudes. There were statistically significant differences among the three groups in gender, education levels, monthly income, patient expected medication management approach and initial medication management approach.

**Table 3:** Description of characteristics by change of attitudes toward SLE.

Characteristics	Levels	Worsen (N = 74)	No change (N = 544)	Better (N = 891)	P value
Sex, n (%)	Man	3 (4.1)	46 (8.5)	48 (5.4)	.049
	Female	71 (95.9)	498 (91.5)	843 (94.6)	
Age (years), mean (SD)	Mean ± SD	34.1 ± 7.7	35.3 ± 11.1	34.5 ± 9.8	.312
Education, n (%)	Primary middle school or below	36 (48.6)	225 (41.4)	261 (29.3)	<.001
	High school	16 (21.6)	144 (26.5)	211 (23.7)	
	Bachelor degree or above	22 (29.7)	175 (32.2)	419 (47)	
Monthly income (¥), n (%)	<3000	47 (63.5)	289 (53.1)	388 (43.5)	<.001
	3000-5000	20 (27.0)	181 (33.3)	324 (36.4)	
	5000-10,000	4 (5.4)	57 (10.5)	138 (15.5)	
	>10,000	3 (4.1)	17 (3.1)	41 (4.6)	
Onset age (years), mean (SD)	Mean ± SD	27.6 ± 7.9	28.8 ± 11.6	28.1 ± 9.8	.437
Chronic diseases before the diagnosis of SLE, n (%)	Yes	10 (13.5)	67 (12.3)	126 (14.1)	.617
	No	64 (86.5)	477 (87.7)	765 (85.9)	
Use of immunosuppressive drugs in the initial medication, n (%)	Yes	59 (79.7)	383 (70.4)	655 (73.5)	.167
	No	15 (20.3)	161 (29.6)	236 (26.5)	
Use of immunosuppressive drugs in the existing medication, n (%)	Yes	62 (83.8)	390 (71.7)	650 (73)	.089
	No	12 (16.2)	154 (28.3)	241 (27)	
Use of biological agents in the initial medication, n (%)	Yes	1 (1.4)	29 (5.3)	47 (5.3)	.322
	No	73 (98.6)	515 (94.7)	844 (94.7)	
Use of biological agents in the existing medication, n (%)	Yes	4 (5.4)	41 (7.5)	63 (7.1)	.791
	No	70 (94.6)	503 (92.5)	828 (92.9)	
Changes in medication, n (%)	No change	47 (63.5)	413 (75.9)	619 (69.5)	.079
	change one time	16 (21.6)	78 (14.3)	179 (20.1)	
	change twice	7 (9.5)	33 (6.1)	62 (7.0)	
	change tripple times	4 (5.4)	20 (3.7)	31 (3.5)	
Development of chronic diseases during treatment, n (%)	No change	60 (81.1)	465 (85.5)	755 (84.7)	.781
	increase one chronic disease	9 (12.2)	57 (10.5)	92 (10.3)	
	increase two chronic diseases	4 (5.4)	13 (2.4)	32 (3.6)	
	increase three chronic diseases or more	1 (1.4)	9 (1.7)	12 (1.3)	
Development of side effects during treatment, n (%)	No change	57 (77.0)	474 (87.1)	752 (84.4)	.065
	increase one extra side effect	16 (21.6)	57 (10.5)	113 (12.7)	
	increase two extra side effects	0 (0)	10 (1.8)	23 (2.6)	
	increase three extra side effects	1 (1.4)	3 (0.6)	3 (0.3)	
Understanding of SLE guideline, n (%)	Not at all	34 (45.9)	244 (44.9)	399 (44.8)	.098
	A little bit	32 (43.2)	196 (36.0)	362 (40.6)	
	Familiar or Understand	8 (10.8)	104 (19.1)	130 (14.6)	
Existing medication management approach, n (%)	Doctor oriented	61 (82.4)	457 (84.0)	765 (85.9)	.419
	Patient involved	10 (13.5)	76 (14.0)	114 (12.8)	
	Self-initiated reduction of medication	3 (4.1)	11 (2.0)	12 (1.3)	
Patient expected medication management approach, n (%)	Patient oriented	2 (2.7)	7 (1.3)	4 (0.4)	.002
	Doctor oriented	50 (67.6)	409 (75.2)	600 (67.3)	
	Patient involved	20 (27.0)	121 (22.2)	278 (31.2)	
	Self-initiated reduction of medication	2 (2.7)	7 (1.3)	9 (1.0)	
Initial medication management approach, n (%)	Doctor oriented	63 (85.1)	483 (88.8)	824 (92.5)	.022
	Patient involved	8 (10.8)	54 (9.9)	57 (6.4)	
	Self-initiated reduction of medication	3 (4.1)	7 (1.3)	10 (1.1)	
Attitude at the phase of diagnosis, n (%)	Negative	0 (0)	68 (12.5)	891 (100.0)	<.001
	Positive	74 (100%)	476 (87.5)	0 (0)	
Attitude from diagnosis to treatment, n (%)	No change	0 (0%)	544 (100.0)	0 (0)	<.001
	Worsen	74 (100%)	0 (0)	0 (0)	
	Better	0 (0%)	0 (0)	891 (100.0)	

**Figure 3:**
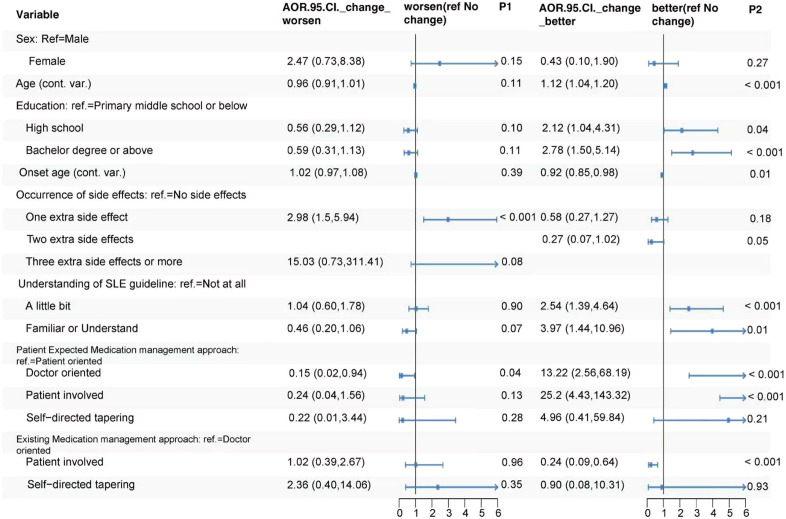
Associations between characteristics and change of attitudes toward SLE.

For changes in attitudes, the following factors showed significantly lower odds of experiencing worsened attitudinal change compared to no change: doctor-oriented medication management expectations versus patient-oriented (aOR = 0.15, 95% CI: 0.02-0.94, *p* = 0.04) (**Figure 3**). Conversely, the occurrence of one extra side effects significantly increased the odds of the worsened attitudinal change (aOR = 2.98, 95% CI: 1.50-5.94, *p* < 0.001). Regarding better attitudinal change, younger onset age (aOR = 0.92, 95% CI: 0.85-0.98, *p* = 0.01) and patient-involved existing medication management compared to doctor-oriented (aOR = 0.24, 95% CI: 0.09-0.64, *p* < 0.001) were associated with significantly lower odds. In contrast, factors significantly associated with higher odds of better attitudinal change included older age (aOR = 1.12, 95% CI: 1.04-1.20, *p*=<0.001), higher education levels of high school (aOR = 2.12, 95% CI: 1.04-4.31, *p* = 0.04) and bachelor’s degree or above vs primary middle school or below (aOR = 2.78, 95% CI: 1.50-5.14, *p* < 0.001), a little bit understanding (aOR = 2.54, 95% CI: 1.39-4.64, *p*=<0.001) or being familiar with the SLE guidelines (aOR = 3.97, 95% CI: 1.44-10.96, *p* = 0.01), doctor-oriented (aOR = 13.22, 95% CI: 2.56-68.19, *p* < 0.001) and patient-involved (aOR = 25.20, 95% CI: 4.43-143.32, *p* < 0.001) medication management expectations compared to patient-oriented (**Figure 3**).

## Discussion

This retrospective study provided important insights into the impact of patients’ attitudes and knowledge on treatment decisions and disease management in patients with SLE. Our findings underscored the critical impact that attitudes toward SLE and understanding of clinical guidelines could have on both initial and sustained treatment decisions. We identified that older age, higher education, guideline familiarity, doctor-oriented and patient-involved management expectations significantly influence better patient attitudinal change, highlighting potential targets for improving patient engagement and clinical outcomes.

At the stage of diagnosis, female patients and those with higher educational backgrounds were less likely to exhibit a positive attitude toward SLE. Such situations suggest that increased awareness and potentially greater expectations among more educated patients might result in greater anxiety or skepticism regarding treatment-related risks and side effects before accepting the treatment. This may be explained by the “information anxiety” phenomenon, where greater health literacy coexists with heightened perception of risks, and by gender-specific psychosocial burdens. Previous studies similarly indicated that heightened disease awareness might not always correlate positively with treatment acceptance due to increased concerns about adverse effects and long-term medication safety (4, 6, 13).Moreover, we observed that familiarity with SLE guidelines significantly improved the likelihood of positive attitudes, indicating the potential role of knowledge of related disease guidelines in alleviating treatment-related anxieties and misconceptions (24). This aligns with the knowledge-attitude-practice (KAP) framework, where knowledge empowerment reduces uncertainty and fosters trust in the treatment pathway (11, 24).

At the stage of post-treatment, attitudes toward SLE notably improved, influenced by factors including higher age, greater educational levels, and clear expectations for doctor-oriented or patient-involved medication management approaches (7, 17). These findings suggest that ongoing engagement with healthcare providers, clear communication, and shared decision-making approaches might considerably enhance patients’ acceptance of and adherence to treatments, ultimately facilitating better chronic disease management and clinical outcomes (17, 19, 20). The preference for collaborative management may enhance treatment ownership and self-efficacy, while structured support reduces anxiety (25). Conversely, our results also suggest that side effects during treatment negatively impacted attitudes, potentially undermining adherence and exacerbating disease progression. This likely operates through increased treatment burden and negative illness perception, where side effects may be interpreted as signs of treatment failure, creating a discouraging feedback loop (21). This highlights the necessity for healthcare providers to proactively manage side effects and effectively communicate risk-benefit profiles to patients throughout treatment (17, 18).

Notably, changes in attitudes (from negative to positive or from positive to negative) highlighted the dynamic nature of attitude toward SLE throughout disease management. Among patients who had better attitudinal change, older age, higher education levels, and stronger familiarity with the SLE guidelines were significantly associated with this transition. Older age may confer more mature coping strategies and life experience, facilitating adaptation, whereas the initial anxiety among the highly educated may be overcome through positive treatment experiences and knowledge consolidation (24). Additionally, patients who expected medication management of doctor oriented or patient involved were substantially more likely to develop positive attitudes. These findings underscore the importance of continuous patient education and enhanced communication strategies as core elements of chronic disease management (14, 20, 26, 27). In contrast, patients who experienced worse attitudinal change were more likely to experience new side effects during treatment and to lack doctor-centered medication guidance. This group suggest that insufficient support during complex or adverse treatment scenarios may contribute to erosion of initial confidence (28, 29). These results highlight the importance of maintaining proactive intervention, active management of side effects, and consistent follow-up to promote positive attitudes toward SLE throughout disease progression, particularly during early stages of treatment.

From a policy and practice perspective, these findings indicate the need for structured educational initiatives targeting patients with lower education levels or limited familiarity with SLE guidelines, particularly older adults. Policies could focus on implementing different training programs for healthcare providers to strengthen doctor-patient communication, improving doctor-guided and patient-involved medication management strategies. Moreover, developing approaches for timely detection and proactive management of side effects may prevent negative shifts in patient attitudes. Healthcare systems could also integrate routine assessments of patient attitudes and experiences into clinical protocols, ensuring continuous identification and support of at-risk patients throughout treatment.

While this study is based on a Chinese cohort - where healthcare structures, socioeconomic landscape, and cultural attitudes toward illness may shape specific findings—the core relationships identified are likely relevant to SLE management globally. For instance, the observed emphasis on physician authority and familial support in medical decisions could influence attitudinal responses in ways that vary across cultures. Nevertheless, the roles of disease knowledge (e.g., guideline familiarity), treatment experience (particularly side effects), and collaborative patient-physician dynamics in shaping illness attitudes reflect challenges common to chronic disease care worldwide. The global emphasis on patient-centered care, shared decision-making, and the psychological impact of chronic disease further supports the broader relevance of these findings. Future studies in diverse healthcare settings are warranted to confirm these associations and to adapt specific interventions locally.

Collectively, our findings, interpreted through potential mechanisms such as knowledge empowerment (11, 24), management of treatment burden (21), and the fostering of self-efficacy through collaborative care (11, 25), underscore that patient attitudes are not static traits but dynamic states shaped by information, experience, and clinical interactions. Future interventions should be phase-specific, aiming to mitigate initial information anxiety, proactively manage treatment burdens like side effects, and consistently foster a supportive and communicative care environment to cultivate and sustain positive attitudes toward SLE management.

## Limitations

Several limitations should be acknowledged in interpreting our results. Firstly, due to the retrospective and observational design, causal relationships could not be firmly established. Second, and most importantly, the assessment of “attitude at diagnosis” relied on patient recall and may have been influenced by current disease status, treatment experiences (e.g., side effects, perceived efficacy), and overall disease understanding. This introduces potential recall bias, which could be outcome−dependent and may affect the estimated magnitude and direction of attitudinal “change” as well as confound associations involving baseline attitudes. Although we controlled for key clinical variables and emphasized the interpretation of the non−retrospective “post−treatment attitude,” the measurement error inherent in recalled attitudes cannot be fully eliminated. Prospective studies with longitudinal follow-ups would better indicate how attitudes and knowledge dynamically influence treatment adherence over time. Third, the study relied heavily on patient self-report, which could introduce recall bias or social desirability bias. Fourth, data collected across multiple healthcare institutions could introduce variability in reporting practices despite standardization efforts. Fifth, as the precise timing of attitude transitions was not captured, we were unable to examine how quickly or gradually these changes occurred during the treatment course. Prospective longitudinal studies are needed to better capture the dynamics of attitude evolution and its influence on treatment adherence over time.

In conclusion, this study maps the attitudinal change toward SLE from diagnosis to post-treatment, and provides critical related factors associated with attitudinal change among Chinese patients with SLE. Our results highlight the importance of enhancing interactions between physicians and patients, ongoing patient education, and proactive intervention and management of treatment. Future research should adopt prospective longitudinal designs to further explore interventions tailored to patient attitudes and knowledge to optimize adherence and improve long-term outcomes for patients with SLE.

## Data Availability

The original contributions presented in the study are included in the article/**Supplementary Material**, Further inquiries can be directed to the corresponding authors.
