# Supplementary material for: Changes in attitudes toward systemic lupus erythematosus and associated factors: a retrospective cross-sectional study from China
Source: Front Immunol. 2026 Jan 26;17:1759777. doi: 10.3389/fimmu.2026.1759777 (PMC12883808; doi:10.3389/fimmu.2026.1759777)
Supplement: Supplementary file 1 [file Table1.docx]

**Supplementary Survey**

1. The hospital you are seeking medical advice is [Fill in the blanks] *

_________________________________

2. What is your gender? [Single Choice] *

| ○ Male |
| --- |
| ○ Female |

3. When were you born? [Fill in the blanks] *

_________________________________

4. What is your height (cm)? [Fill in the blanks] *

_________________________________

5. What is your height (weight)? [Fill in the blanks] *

_________________________________

6. Where is your registered permanent residence? [Fill in the blanks] *

_________________________________

7. Where is your current place of work or residence (lived for more than six months)? [Fill in the blanks] *

_________________________________

8. What is your educational level? [Single Choice] *

| ○ Junior high school or lower |
| --- |
| ○ Senior high school |
| ○ College or higher |

9. Which income class are you (RMB/month)? [Single Choice] *

| ○ <3000 |
| --- |
| ○ 3000-5000 |
| ○ 5000-10000 |
| ○ 10000-20000 |
| ○ >20000 |

10. When were you first diagnosed with systemic lupus erythematosus (SLE)? [Fill in the blanks] *

_________________________________

11. Were you diagnosed with lupus nephritis (LN)? [Single Choice] *

| ○No |
| --- |
| ○ Yes, the time of diagnosis of LN was the same as SLE |
| ○ Yes, the time of diagnosis of LN was not the same as SLE |

12. When were you first diagnosed with LN? [Fill in the blanks] *

_________________________________

13. (For patients with LN development) Have you ever been in the following situation? [Multiple Choice] *

| □ Arbitrary reductions of dose |
| --- |
| □ Irregular treatment |
| □ Drug resistance |
| □ Others _________________* |

14. (For patients with LN) Did you have a kidney biopsy? [Single Choice] *

| ○ Yes |
| --- |
| ○ No |

15. What is your histologic classification of nephritis? [Single Choice] *

| ○ Class Ⅰ: minimal mesangial LN |
| --- |
| ○ Class Ⅱ: mesangial proliferative LN |
| ○ Class Ⅲ: focal LN |
| ○ Class Ⅳ: diffuse LN |
| ○ Class Ⅴ: membranous LN |
| ○ Class Ⅵ: advanced sclerosing LN |
| ○ Class Ⅲ+ Class Ⅴ |
| ○ Class Ⅳ+ Class Ⅴ |

16. What is the current stage of the disease? [Single Choice] *

| ○ Stable |
| --- |
| ○ Active |

17. What chronic disease(s) did you have before the diagnosis of SLE? [Multiple Choice, several answers possible] *

| □ None |
| --- |
| □ Hypertension |
| □ Dyslipidemia |
| □ Diabetes |
| □ Tumor |
| □ Chronic lung disease |
| □ Stroke |
| □ Liver disease |
| □ Chronic gastritis |
| □ Cardiovascular disease |
| □ Chronic bronchitis |
| □ Others _________________* |

18. Since the onset, have you reached a stable period (or clinical remission) through treatment? [Single Choice] *

| ○ Yes |
| --- |
| ○ No |

19. If you have reached a stable period (clinical remission), have you ever relapsed? [Single Choice] *

| ○ Yes |
| --- |
| ○ No |

20. (For patients who experienced relapse) Have you ever been in the following situation? [Single Choice, several answers possible] *

| □ Arbitrary reductions of dose |
| --- |
| □ Irregular treatment |
| □ Drug resistance |
| □ Others _________________* |

21. Are you currently using hydroxychloroquine for treatment? [Single Choice] *

| ○ Yes，the dose is (g/day) or (tablet/day) _________________ * |
| --- |
| ○ No |

22. Are you currently using glucocorticoids for treatment? [Single Choice] *

| ○ Yes，the dose is (g/day) or (tablet/day) _________________ * |
| --- |
| ○ No |

23. In addition to hydroxychloroquine and glucocorticoids, which of the following medications are you currently using: [Multiple Choice, several answers possible] *

| □ Cyclophosphamide |
| --- |
| □ Mycophenolate mofetil |
| □ Azathioprine |
| □ Rituximab |
| □ Tacrolimus |
| □ Cyclosporine |
| □ Methotrexate |
| □ Belimumab |
| □ Plasma exchange/continuous plasma filtration absorption |
| □ Others _________________* |
| □ None |

24. If you remember, your current dose of the drug is: [Multiple Choice, several answers possible]

| □ Cyclophosphamide, the dose is (mg/month) _________________* |
| --- |
| □ Mycophenolate mofetil, the dose is (mg/day) _________________* |
| □ Azathioprine, the dose is (mg/day) _________________* |
| □ Rituximab , the dose is (mg/month) _________________* |
| □ Tacrolimus, the dose is (mg/day) _________________* |
| □ Cyclosporine, the dose is (mg/day) _________________* |
| □ Methotrexate, the dose is (mg/day) _________________* |
| □ Belimumab, the dose is (mg/month) _________________* |
| □ Plasma exchange/continuous plasma filtration absorption |
| □ Other, the dose is _________________* |
| □ None |

25. Has your current treatment been up to six months? [Single Choice] *

| ○ Yes |
| --- |
| ○ No |

26. How many months has been your current treatment plan? [Fill in the blanks] *

_________________________________

27. Do you currently have proteinuria? [Single Choice] *

Proteinuria refers to the urine protein content in the 24-hour urine sediment being greater than 0.5g, or the urine protein-creatinine ratio being less than 0.5mg/mmol during routine urine examination

| ○ Yes, the value is (g/24-hour) _________________ |
| --- |
| ○ No |

28. If you remember, your current blood creatinine value (μmol/l) is [Fill in the blanks]

_________________________________

29. Have you ever changed immunosuppressive drugs? [Single Choice] *

| ○ No (Please skip to question 34) |
| --- |
| ○ Changed once |
| ○ Changed twice |
| ○ Changed three times or more |

30. When did you change to the current treatment plan? [Fill in the blanks] *

_________________________________

31. The medication you use for the first time is: [Multiple Choice, several answers possible] *

| □ Cyclophosphamide |
| --- |
| □ Mycophenolate mofetil |
| □ Azathioprine |
| □ Rituximab |
| □ Tacrolimus |
| □ Cyclosporine |
| □ Methotrexate |
| □ Belimumab |
| □ Others _________________* |

32. Which immunosuppressive drug(s) and biological agents(s) have you replaced later [Multiple Choice, several answers possible] *

| □ Cyclophosphamide |
| --- |
| □ Mycophenolate mofetil |
| □ Azathioprine |
| □ Rituximab |
| □ Tacrolimus |
| □ Cyclosporine |
| □ Methotrexate |
| □ Belimumab |
| □ Others _________________* |

33. What caused you to change your treatment plan? [Multiple Choice, several answers possible] *

| □ Efficacy |
| --- |
| □ Relapse |
| □ Side effects (what kind of side effects) _________________* |
| □ Impact of chronic disease (what kind of chronic disease) _________________* |
| □ Treatment price |
| □ Voluntary adjustment of medication |
| □ Other _________________* |

34. Is the hospital where you were diagnosed and treated for the first time the same as the current one? [Single Choice] *

| ○ Yes |
| --- |
| ○ No, the hospital at the time of first treatment is _________________ * |

35. From the diagnosis of the disease to treatment, how many types of chronic diseases have you developed? [Single Choice] *

| ○ No |
| --- |
| ○ Developed one chronic disease |
| ○ Developed two or more chronic diseases |

36. The chronic disease(s) you have developed is/are: [Multiple Choice, several answers possible] *

| □ None |
| --- |
| □ Hypertension |
| □ Dyslipidemia |
| □ Diabetes |
| □ Tumor |
| □ Chronic lung disease |
| □ Stroke |
| □ Liver disease |
| □ Chronic gastritis |
| □ Cardiovascular disease |
| □ Chronic bronchitis |
| □ Others _________________* |

37. Do you have any serious drug-related adverse events? [Single Choice] *

| ○ No |
| --- |
| ○ Developed one chronic disease |
| ○ Developed two or more chronic diseases |

38. The adverse event(s) you have developed is/are: [Multiple Choice, several answers possible] *

| □ Femoral head necrosis |
| --- |
| □ Retinal macular degeneration |
| □ Cataract |
| □ Glaucoma |
| □ Serious infection |
| □ Others _________________* |

39. Do you know the "2020 Chinese guidelines for the diagnosis and treatment of systemic lupus erythematosus"? [Single Choice] *

| ○ Not familiar at all |
| --- |
| ○ Familiar (simply familiar with some items of the guidelines) |
| ○ Very familiar (Have carefully read relevant treatment contents, and can choose a treatment plan according to the health condition) |

40. The way you receive the current medication is: [Single Choice] *

| ○ Choose according to the treatment guidelines |
| --- |
| ○ Follow the doctor's advice completely |
| ○ Discuss with the doctor |
| ○ Self-discontinuation or reduction of medication |

41. The way you receive the first medication was: [Single Choice] *

| ○ Choose according to the guidelines |
| --- |
| ○ Follow the doctor's advice completely |
| ○ Discuss with the doctor |
| ○ Self-discontinuation or reduction of medication |

42. In the future, the way you will receive the medication tend to： [Single Choice] *

| ○ Choose according to the guidelines |
| --- |
| ○ Follow the doctor's advice completely |
| ○ Discuss with the doctor |
| ○ Self-discontinuation or reduction of medication |

43. How has your attitude towards the disease changed from diagnosis to treatment? [Single Choice] *

| ○ From fear to fear |
| --- |
| ○ From fear to acceptance |
| ○ From acceptance to fear |
| ○ From acceptance to acceptance |

44. Do you think the change in your attitude impacts the prognosis of the disease? [Single Choice] *

| ○Yes |
| --- |
| ○ No |

45. What do you think your attitude has had on the prognosis of the disease? [Single Choice, several answers possible] *

| □ Efficacy of the drug |
| --- |
| □ Side effects |
| □ Relapse |
| □ Others (brief description) _________________* |

46. Do you think the reason(s) for the change in your attitude is/are: [Multiple Choice, several answers possible] *

| □ Efficacy of the drug |
| --- |
| □ Side effects |
| □ Growth of age |
| □ Personal ideals or beliefs |
| □ Love and encouragement from friends |
| □ The care or responsibility of the family |
| □ Social care or responsibility |
| □ Expectations for new drugs |
| □ Others (brief description) _________________* |

47. Have you given birth? [Single Choice] *

| ○ Yes |
| --- |
| ○ No |

48. Did you have a child plan at the time of diagnosis? [Single Choice] *

| ○ Yes |
| --- |
| ○ No |

49. Do you think SLE will affect your intention to have children in the future? [Single Choice] *

| ○ Yes |
| --- |
| ○ No |

50. What is your name? [Fill in the blanks] *

_________________________________

51. What is your phone number? [Fill in the blanks] *

_________________________________
